# Modelling continuous abstinence rates over time from clinical trials of pharmacological interventions for smoking cessation

**DOI:** 10.1111/add.14549

**Published:** 2019-01-29

**Authors:** Sarah E. Jackson, Jennifer A. McGowan, Harveen Kaur Ubhi, Hannah Proudfoot, Lion Shahab, Jamie Brown, Robert West

**Affiliations:** ^1^ Department of Behavioural Science and Health University College London London UK

**Keywords:** Bupropion, continuous abstinence, nicotine replacement therapy, pharmacological interventions, relapse, smoking cessation, smoking cessation aids, varenicline

## Abstract

**Background and aim:**

It is useful, for theoretical and practical reasons, to be able to specify functions for continuous abstinence over time in smoking cessation attempts. This study aimed to find the best‐fitting models of mean proportion abstinent with different smoking cessation pharmacotherapies up to 52 weeks from the quit date.

**Methods:**

We searched the Cochrane Database of Systematic Reviews to identify randomized controlled trials (RCTs) of pharmacological treatments to aid smoking cessation. For comparability, we selected trials that provided 12 weeks of treatment. Continuous abstinence rates for each treatment at each follow‐up point in trials were extracted along with methodological details of the trial. Data points for each pharmacotherapy at each follow‐up point were aggregated where the total across contributing studies included at least 1000 participants per data point. Continuous abstinence curves were modelled using a range of different functions from the quit date to 52‐week follow‐up. Models were compared for fit using *R* ^2^ and Bayesian information criterion (BIC).

**Results:**

Studies meeting our selection criteria covered three pharmacotherapies [varenicline, nicotine replacement therapy (NRT) and bupropion] and placebo. Power functions provided the best fit (*R* ^2^ > 0.99, BIC < 17.0) to continuous abstinence curves from the target quit date in all cases except for varenicline, where a logarithmic function described the curve best (*R* ^2^ = 0.99, BIC = 21.2). At 52 weeks, abstinence rates were 22.5% (23.0% modelled) for varenicline, 16.7% (16.0% modelled) for bupropion, 13.0% (12.4% modelled) for NRT and 8.3% (8.9% modelled) for placebo. For varenicline, bupropion, NRT and placebo, respectively, 55.9, 65.0, 62.3 and 56.5% of participants who were abstinent at the end of treatment were still abstinent at 52 weeks.

**Conclusions:**

Mean continuous abstinence rates up to 52 weeks from initiation of smoking cessation attempts in clinical trials can be modelled using simple power functions for placebo, nicotine replacement therapy and bupropion and a logarithmic function for varenicline. This allows accurate prediction of abstinence rates from any time point to any other time point up to 52 weeks.

## Introduction

Continuous abstinence rates after initiation of a smoking quit attempt follow a negatively decelerating curve over time 1. This is a common pattern with attempts to stop engaging in addictive behaviours 2. It is useful to be able to model the curve relating continuous abstinence to time from quit attempt initiation in order to understand the processes underlying smoking relapse and the impact of treatments on these processes, as well as for the more practical goal of predicting long‐term abstinence rates from short‐term outcomes. This paper synthesizes evidence from clinical trials of pharmacotherapies in smoking cessation to derive best‐fitting, up‐to‐date models of mean continuous abstinence rates as a function of time since the quit attempt started.

Results of clinical trials and cohort studies suggest that with unaided quit attempts, only approximately a quarter of those trying to quit remain abstinent for a week, fewer than one in 10 remain abstinent for 6 months and fewer than one in 20 remain abstinent at 1 year 1. Continuous abstinence curves for smokers receiving support for quitting can be derived for a number of individual studies with multiple follow‐up points 3, 4, 5, but functions describing the shape of the continuous abstinence curve over time have not been specified thus far.

Some studies have specifically addressed the question of whether relapse rates increase when treatment is terminated. A recent synthesis of evidence from trials of varenicline showed a higher relapse rate from the end of 12 weeks of treatment to 24‐week follow‐up than for placebo 6, but the difference had disappeared by 52‐week follow‐up. Studies exploring the long‐term impact of nicotine replacement therapy (NRT) have found diminishing efficacy in terms of absolute percentage differences from placebo with increased time since quit attempt initiation 7, 8. None of these studies sought to model the shape of the relapse curve, however.

Modelling the shape of continuous abstinence curves in smoking cessation is important for several reasons: (1) if the continuous abstinence curves follow a well‐defined and relatively simple mathematical formula, it would allow for prediction of abstinence rates from any time‐point to any future time‐point, which is important clinically and when assessing health impact 9. (2) The shape of the curve may help in understanding what drives resumption of smoking at different time‐points. It has been suggested that factors affecting early resumption of smoking may differ in kind from those driving later resumption 10. This, in turn, has implications for interventions that may be effective at different time‐points. (3) If the curves follow different shapes with different pharmacotherapies, it suggests that these may operate differentially on factors influencing resumption of smoking. For example, if the curve shows an inflection downwards post‐treatment for a given pharmacotherapy, it suggests either that it is not adequately addressing chronic factors that lead to smoking resumption or that the treatment duration was too short.

When seeking to study continuous abstinence curves it is important to have high‐quality data with high follow‐up rates. Prospective cohort studies can provide valuable information, but often there are limitations with regard to loss to follow‐up and inconsistencies in treatment duration that make it difficult to make meaningful comparisons. With greater control over treatment conditions, higher retention rates and data collection occurring at multiple time‐points, randomized controlled trials (RCTs) provide an opportunity to examine continuous abstinence following pharmacological interventions for smoking cessation in greater detail. This study therefore aggregated data from high‐quality RCTs to describe the shape and parameters of mean continuous abstinence curves associated with different smoking cessation pharmacotherapies. Specifically, we aimed to address the following research questions:

1. What functions best characterize the shape of mean continuous abstinence curves over the first year following quit attempts in smoking cessation RCTs?
2. Do these differ with different pharmacotherapies?

## Method

### Search strategy and study selection

**Figure 1:**
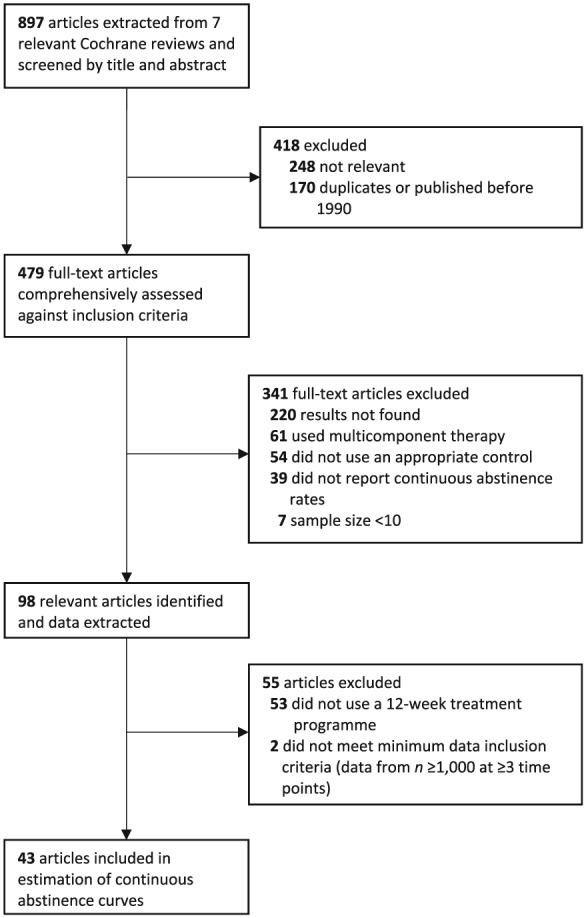
Flow diagram of search strategy and study selection

Details of our study search and selection procedure are provided in Fig. 1. We searched the Cochrane Database of Systematic Reviews in the Cochrane Library (from 1990 to March 2017) for reviews with ‘smoking’ in the title, abstract or keyword fields. The results were assessed by three authors (J.M., H.U., R.W.) in order to identify reviews focusing on pharmacological treatments for smoking cessation; seven were identified as being appropriate for this study 11, 12, 13, 14, 15, 16, 17. The reference lists of these reviews provided a pool of potential studies for inclusion in the present study. Supporting information, Appendix S1 contains an Excel file providing details of all studies considered in case readers wish to undertake their own analyses.

We included RCTs that compared continuous abstinence rates between pharmacological interventions, between one intervention and a placebo or between different pharmacotherapy doses and a placebo. We limited our search to articles published since 1990 because we considered trial reporting and conduct to have improved since then, with agreed reporting criteria for trials such as Consolidated Standards of Reporting Trials (CONSORT) being more widely used 18. Trials that compared a pharmacological intervention with ‘standard care’ were excluded, as were studies that focused on a reduction in cigarette consumption, or setting of quit dates rather than abstinence. We also excluded trials of combinations of different pharmacotherapies. Trials that reported only point prevalence (as opposed to continuous) abstinence rates were excluded, because it was not possible to determine whether abstinence was the result of the initial quit attempt. Trials with a sample size < 10 participants were also excluded, because it was judged that these would have been pilot or feasibility trials. We did not exclude any studies by virtue of heaviness of smoking or other smoker characteristics, but the large majority of studies set a minimum of 10 or more cigarettes per day for inclusion.

Due to the stringent criteria of Cochrane Reviews, all included RCTs were considered to be of acceptable standard. Studies awaiting assessments and ongoing studies were included where results were available.

### Data extraction

RCTs that met the criteria detailed above were extracted from the relevant Cochrane Reviews. From these trials we extracted information on sample size, pharmacotherapies used, length of pharmacotherapy intervention, start point of follow‐up, duration of follow‐up and the percentage of participants who were continuously abstinent over time in each condition from a given start point to each follow‐up point. Other data extracted from the trials included year of publication, year of trial start and country of origin.

### Analyses

There was significant heterogeneity across study samples, methods and presentation of results. In order to standardize results for comparison across different pharmacotherapies while maximizing data available for the estimation of continuous abstinence curves, we selected studies that had used a 12‐week treatment programme; this was the modal and median duration of treatments in the 98 studies 4, 5, 19, 20, 21, 22, 23, 24, 25, 26, 27, 28, 29, 30, 31, 32, 33, 34, 35, 36, 37, 38, 39, 40, 41, 42, 43, 44, 45, 46, 47, 48, 49, 50, 51, 52, 53, 54, 55, 56, 57, 58, 59, 60, 61, 62, 63, 64, 65, 66, 67, 68, 69, 70, 71, 72, 73, 74, 75, 76, 77, 78, 79, 80, 81, 82, 83, 84, 85, 86, 87, 88, 89, 90, 91, 92, 93, 94, 95, 96, 97, 98, 99, 100, 101, 102, 103, 104, 105, 106, 107, 108, 109, 110, 111, 112, 113, 114, 115 from which we extracted data. We used Microsoft Excel 2013 to plot the mean continuous abstinence rates weighted by sample size for follow‐up points where there were aggregated data from a minimum of 1000 participants for all treatment groups. The resulting graphs describe the mean continuous abstinence rates associated with the different pharmacotherapies, displaying curves from the start of treatment to 52 weeks (the longest follow‐up for which the required data were available). For each treatment group, we compared four function classes (linear, exponential, logarithmic, and power) and fitted the one with the highest *R* ^2^. To handle zero values in the case of power functions, the starting time‐point was 0.1 week, representing less than 1 day of abstinence. Model selection was confirmed by comparing the Bayesian information criterion (BIC) of each curve (calculated in Stata version 15), with lower BIC values indicating better model fit.

To check how far the relapse curves from the aggregated data matched within‐study changes in continuous abstinence rates over time, we superimposed lines between consecutive pairs of time‐points with each of the three largest studies, including where there were data available for additional time‐points for each pharmacotherapy, onto our continuous abstinence curves to assess whether the curves were a reasonable fit.

## Results

Our search and selection procedure (Fig. 1) identified 43 trials eligible for inclusion in the present analysis. Their characteristics are summarized in Table 1. A total of 23 trials used varenicline 4, 69, 70, 71, 74, 77, 78, 80, 84, 85, 86, 87, 88, 89, 91, 93, 95, 98, 101, 103, 109, 110, 115, 12 used bupropion 4, 50, 56, 63, 64, 66, 69, 70, 85, 90, 95, 101 and 15 used NRT 4, 22, 25, 28, 29, 30, 32, 48, 49, 72, 88, 90, 100, 105, 113, with some studies using more than one of these pharmacotherapies in different treatment groups. A placebo was used as a control in 38 of these trials 4, 22, 25, 28, 29, 30, 32, 48, 49, 50, 56, 63, 64, 66, 69, 70, 71, 72, 74, 77, 78, 84, 85, 86, 87, 89, 91, 93, 95, 98, 100, 101, 103, 105, 109, 110, 113, 115, and a further two trials provided only placebo data due to the active comparator failing to meet criteria for inclusion 45, 104.

**Table 1:** Characteristics of included studies.

Authors	Year	Country of origin	Special samples	Pharmacotherapies (italics indicate those not included in modelling)	n (in order of stated pharmacotherapies)	Follow‐up points (weeks)
Sachs *et al*. 22	1993	Sweden	–	NRT patch, placebo	110, 110	6, 12, 18, 26, 52
Imperial Cancer Research fund General practice research group 25	1994	UK	–	NRT patch, placebo	842, 844	12, 52
Gourlay *et al*. 29	1995	Australia	–	NRT patch, placebo	315, 314	4, 8, 12, 26
Stapleton *et al*. 28	1995	UK	–	NRT patch, placebo	800, 400	3, 6, 12, 26, 52
Campbell *et al*. 30	1996	UK	Hospital patients	NRT patch, placebo	115, 119	12, 52
Sønderskov *et al*. 32	1997	Denmark	–	NRT patch, placebo	251, 142	4, 8, 12, 26
Wong *et al*. 45	1999	USA	–	*Naltrexone*, placebo	23, 26	1, 2, 3, 4, 6, 8, 10, 12, 24
Tønnesen *et al*. 49	2000	Denmark	Lung clinic attenders	NRT patch, NRT inhaler, placebo	104, 118, 109	2, 6, 12, 36, 52
Wallström *et al*. 48	2000	Sweden	–	NRT sublingual tablet, placebo	123, 124	6, 12, 24, 52
Tashkin *et al*. 50	2001	USA	Chronic obstructive pulmonary disease	Bupropion, placebo	129, 149	5, 6, 7, 10, 12, 26
Trial ZYB40001 56	2003	Canada	–	Bupropion, placebo	141, 143	7, 12
Evins *et al*. 64	2005	USA	Schizophrenics	Bupropion, placebo	25, 28	12
Wagena *et al*. 63	2005	Netherlands	At risk for chronic obstructive pulmonary disease	Bupropion, *nortriptyline*, placebo	86, 80, 89	12, 26
Gonzales *et al*. 69	2006	USA	–	Varenicline, bupropion, placebo	352, 329, 344	12, 24, 52
Jorenby *et al*. 70	2006	USA	–	Varenicline, bupropion, placebo	344, 342, 341	12, 24, 52
Oncken *et al*. 71	2006	USA	–	Varenicline, placebo	518, 129	7, 12, 24, 52
Rigotti *et al*. 66	2006	USA	Hospitalized with acute cardiovascular disease	Bupropion, placebo	124, 123	12, 52
David *et al*. 72	2007	UK	–	NRT patch, placebo	370, 371	12, 24
Nakamura *et al*. 74	2007	Japan	–	Varenicline, placebo	465, 154	12, 24, 52
Tsai *et al*. 77	2007	Korea, Taiwan	–	Varenicline, placebo	126, 124	12, 24
Aubin *et al*. 80	2008	UK, USA, Belgium, France, Netherlands	–	Varenicline, *NRT patch* a	376, 370	12, 52
Niaura *et al*. 78	2008	USA	–	Varenicline, placebo	157, 155	7, 12, 24, 52
Wang *et al*. 84	2009	China, Singapore, Thailand	–	Varenicline, placebo	158, 161	12, 24
Fagerström *et al*. 87	2010	Norway, Sweden	–	Varenicline, placebo	213, 218	12, 26
Fagerström *et al*. 89	2010	6 Asian countries	–	Varenicline, placebo	447, 446	12, 24
Hays *et al*. 85	2010	USA	–	Varenicline, bupropion, placebo	692, 669, 684	12
Rigotti *et al*. 86	2010	15 countries	Stable cardiovascular disease	Varenicline, placebo	355, 359	12, 24, 52
Tsukahara *et al*. 88	2010	Japan	–	Varenicline, NRT patch	16, 16	12, 24
Bolliger *et al*. 93	2011	11 countries	–	Varenicline, placebo	390, 198	12, 24
Tashkin *et al*. 91	2011	USA, Spain, France, Italy	Mild/moderate chronic obstructive pulmonary disease	Varenicline, placebo	250, 254	12, 24, 52
Wittchen *et al*. 90	2011	Germany	–	Bupropion, NRT	108, 105	52
Xenakis *et al*. 95	2011	USA	–	Varenicline, bupropion, placebo	696, 671, 685	12, 52
Rennard *et al*. 98	2012	14 countries	–	Varenicline, placebo	493, 166	12, 24
Tønnesen *et al*. 100	2012	Denmark, Germany	–	NRT mouth spray, placebo	318, 161	4, 6, 8, 12, 16, 20, 24, 52
Anthenelli *et al*. 103	2013	8 countries	Major depression	Varenicline, placebo	256, 269	12, 24, 52
Bullen *et al*. 105	2013	New Zealand	–	NRT patch, *e‐cigarettes*, placebo	295, 289, 73	4, 12, 24
Caponnetto *et al*. 104	2013	Italy	–	*E‐cigarettes*, placebo	200, 100	12, 52
Cinciripini *et al*. 101	2013	USA	–	Varenicline, bupropion, placebo	86, 102, 106	12, 24, 36
Gonzales *et al*. 110	2014	8 countries	–	Varenicline, placebo	249, 245	12, 24, 52
Trial NCT01347112 109	2014	USA	Alcoholics	Varenicline, placebo	16, 17	12, 24
O'Brien *et al*. 113	2015	New Zealand	With and without mental illness	NRT patch, *e‐cigarettes*, placebo	260, 250, 61	24
Anthenelli *et al*. 4	2016	16 countries	With and without psychiatric disorders	Varenicline, bupropion, NRT patch, placebo	2037, 2034, 2038, 2035	12, 24
Eisenberg *et al*. 115	2016	Canada	Hospitalized patients with acute coronary syndrome	Varenicline, placebo	151, 151	4, 12, 24

All studies involved 12 weeks of pharmacotherapy.

a Nicotine replacement therapy (NRT) patch was administered for 10 weeks. so results were not included in analyses.

**Figure 2:**
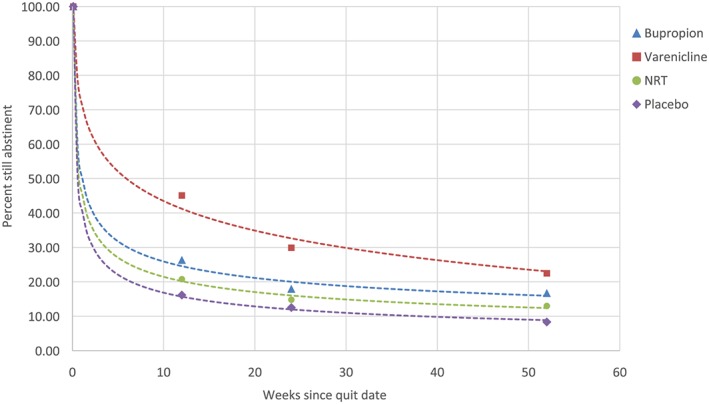
Continuous abstinence rates with best‐fitting curves estimated from continuous abstinence rates from randomized controlled trials (RCTs) of 12‐week treatment with smoking cessation pharmacotherapies. [Colour figure can be viewed at wileyonlinelibrary.com]

Figure 2 shows the continuous abstinence curves from the quit date to 52‐week follow‐up for varenicline, bupropion, NRT and placebo. An interactive version of this graph is available in Supporting information, Appendix S2. The shape of the continuous abstinence curve was similar throughout the different pharmacotherapies (including placebo), with relapse rates highest in the initial 3–4 weeks and slowly tapering off after the end of treatment. Comparison of model selection statistics (Table 2) indicated that continuous abstinence data for bupropion, NRT and placebo could best be described by a power function (all *R* ^2^ > 0.99), while the best‐fitting curve for varenicline was logarithmic (*R* ^2^ = 0.99). Equations for the best‐fitting curve for each pharmacotherapy are shown in Fig. 2. The power function for varenicline also fitted well (Supporting information, Fig. S1: *R* ^2^ = 0.94), but it overestimated the 52‐week abstinence rate.

**Table 2:** Model selection statistics for continuous abstinence curves from 0 to 52 weeks.

	Varenicline		Bupropion		NRT		Placebo	
	R ^2^	BIC	R ^2^	BIC	R ^2^	BIC	R ^2^	BIC
Linear	0.669	37.01	0.516	39.60	0.499	40.12	0.503	40.44
Exponential	0.817	31.53	0.619	31.97	0.613	30.73	0.678	28.78
Logarithmic	0.994	21.16	0.984	25.88	0.980	27.23	0.980	27.55
Power	0.940	26.69	0.991	16.92	0.996	11.75	0.998	7.29

BIC = Bayesian information criterion; NRT = nicotine replacement therapy.

At the end of treatment, 45.1% (41.2% modelled; logarithmic curve) of participants treated with varenicline, 26.3% (24.6% modelled) of those treated with bupropion, 20.8% (20.2% modelled) of those treated with NRT and 16.2% (15.7% modelled) of those treated with placebo were still abstinent. By 52 weeks, abstinence rates had fallen to 22.5% (23.0% modelled) for varenicline, 16.7% (16.0% modelled) for bupropion, 13.0% (12.4% modelled) for NRT and 8.3% (8.9% modelled) for placebo. A total of 49.8% (55.9% modelled) of those treated with varenicline, 63.4% (65.0% modelled) of those treated with bupropion, 62.5% (61.3% modelled) of those treated with NRT and 51.5% (56.5% modelled) of those treated with placebo who were abstinent at the end of treatment were still abstinent at 52 weeks.

Superimposing lines drawn between continuous abstinence rates from consecutive follow‐up points within the three largest studies for each pharmacotherapy showed a good fit to the modelled curves (Supporting information, Fig. S2).

## Discussion

There were sufficient data to enable reliable estimation of mean continuous abstinence rates for end of treatment (12‐week post‐quit date), 26‐ and 52‐week time‐points. The shape of the function relating these to time could best be modelled by power functions for placebo, NRT and bupropion and a logarithmic function for varenicline.

Being able to describe continuous abstinence curves using a simple function, in theory, provides a basis for predicting relapse from one time‐point to any other time‐point. This is useful when one only has short‐term follow‐up data available, or when interpolating back from long‐term data. In UK stop‐smoking services, for example, it is common to use 4‐ or 12‐week abstinence rates in targets because limited resources make it impracticable to collect accurate longer‐term data. Unfortunately, there were not sufficient data to estimate continuous abstinence rates prior to 12 weeks, so it is possible that more complex functions during the treatment period may have been missed. However, we were able to provide precise estimates of mean relapse rates following end of treatment for the different pharmacotherapies.

The finding that a single function starting at the quit date was able to capture abstinence rates at the end of treatment, as well as 26‐ and 52‐week abstinence rates, suggests that a single relapse process is in operation during and after treatment. This appears to conflict with studies suggesting that different processes may be in operation. However, it remains possible that different processes are in operation, but their combined effects remain the same. This is a topic that merits further investigation.

We did not see evidence for a point of inflection in the curves at the end of treatment with the active pharmacotherapies, which also suggests that by 12 weeks from the quit date these pharmacotherapies have had whatever effect they are going to have. This accords with findings from trials with NRT which have not found clear evidence for a benefit of extending treatment beyond 12 weeks 5, 7. It conflicts with findings from a large RCT with varenicline, which showed a benefit from extending treatment for a further 12 weeks 116. However, this benefit appeared to occur with participants who had not managed to attain abstinence early on and had only been abstinent for a short while when the 12‐week course of treatment ended 117. This suggests an optimum treatment regimen for varenicline of 12 weeks if smokers are able to abstain during the first week, but 24 weeks if they only manage to abstain after a few weeks—the aim being to ensure that they receive approximately 12 weeks of pharmacotherapy from the point at which they initiate abstinence.

Of the four functions we fitted to the data, power functions provided the best fit for continuous abstinence from all treatments with the exception of varenicline, which was better described by a logarithmic curve. This difference was driven by the higher 12‐week abstinence rate observed in participants treated with varenicline. Methodological differences between trials of varenicline and other pharmacotherapies may account for at least some of this variance. Varenicline trials typically measure continuous abstinence from week 9 rather than from the target quit date, so the figure may be somewhat inflated, given that varenicline recruits smokers into abstinence for several weeks after the target quit date 118. The results suggest that there is no benefit in recruiting these additional smokers into abstinence, as they are more likely to relapse between the end of treatment and 52‐week follow‐up compared with those treated with other pharmacotherapies.

This study had several limitations. First, there was substantial heterogeneity in trial methods and study samples which may have introduced noise to the data points. Secondly, there were only sufficient data to plot abstinence rates for 12, 24 and 52 weeks after the start of treatment. It would have been useful to have data available on a greater number of time‐points to incorporate into our continuous abstinence curves, particularly in the early weeks where relapse rates were very high. Thirdly, we were only able to include three pharmacotherapies and placebo in our analysis. There were insufficient data available on combination NRT and other popular and emerging pharmacological aids to smoking cessation, such as e‐cigarettes, nortriptyline and cytisine, and it is possible that the continuous abstinence curves associated with these treatments may differ from those observed here. Fourthly, we pre‐specified a small number of functions, and it may be that another function would be more appropriate. Although the fit of the selected function was very high, there was only a small number of follow‐up points. With more follow‐up points, a better‐fitting function might emerge. Fifthly, while a power function provided a very good fit in most cases, the *y* value is infinite at *x* = 0 rather than 100%, so the starting point for time has to be a number above 0. We chose 0.1 weeks (representing less than 1 day) as a value close to 0, and this provided a very close fit to the data. However, a more mathematically sound function with a similar shape may be preferable. Finally, we limited follow‐up to 52 weeks because of the scarcity of data points after that point. Studies with longer‐term follow‐up have been conducted, and these suggest that approximately 30% of those who abstain for 52 weeks relapse at some point within the next 10 years 7. This suggests that the power function, which has a very shallow slope after 52 weeks, may be an adequate fit to the longer‐term continuous abstinence curve.

In conclusion, this study indicates that a power function, or a function very close to it in shape, provides a very close fit to mean continuous abstinence from smoking in studies of smokers trying to quit using placebo, nicotine replacement therapy or bupropion. For varenicline, a logarithmic function appears to provide a better fit.

## Declaration of interests

J.B. has received unrestricted research funding from Pfizer, who manufacture smoking cessation medications. L.S. has received a research grant and honoraria for a talk and travel expenses from manufacturers of smoking cessation medications (Pfizer and Johnson & Johnson). R.W. undertakes research and consultancy for and receives travel funds and hospitality from manufacturers of smoking cessation medications (Pfizer, GlaxoSmithKline and Johnson and Johnson). All authors declare no financial links with tobacco companies or e‐cigarette manufacturers or their representatives.

## Supporting information


**Appendix S1** Excel file containing details of all studies considered for inclusion.

**Appendix S2** Interactive graph showing abstinence rates with best‐fit curves estimated from continuous abstinence rates from RCTs of 12‐week treatment with smoking cessation pharmacotherapies.

**Figure S1** Continuous abstinence rates with power curve for varenicline estimated from continuous abstinence rates from RCTs of smoking cessation.
**Figure S2** Continuous abstinence rates with best‐fit curves estimated from continuous abstinence rates from RCTs of 12‐week treatment with (A) bupropion, (B) varenicline, (C) nicotine replacement therapy and (D) placebo, with consecutive pairs of data points from the three largest studies for each treatment.
