# Supplementary material for: Modelling continuous abstinence rates over time from clinical trials of pharmacological interventions for smoking cessation
Source: Addiction. 2019 Jan 29;114(5):787–97. doi: 10.1111/add.14549 (PMC6492005; doi:10.1111/add.14549)
Supplement: Supplementary file 3 — Figure S1 Continuous abstinence rates with power curve for varenicline estimated from continuous abstinence rates from RCTs of smoking cessation. Figure S2 Continuous abstinence rates with best‐fit curves estimated from continuous abstinence rates from RCTs of 12‐week treatment with (A) bupropion, (B) varenicline, (C) nicotine replacement therapy and (D) placebo, with consecutive pairs of data points from the three largest studies for each treatment. [file ADD-114-787-s002.pdf]

Supplementary Figure 1. Continuous abstinence rates with power curve for varenicline estimated from continuous abstinence rates from RCTs of smoking cessation

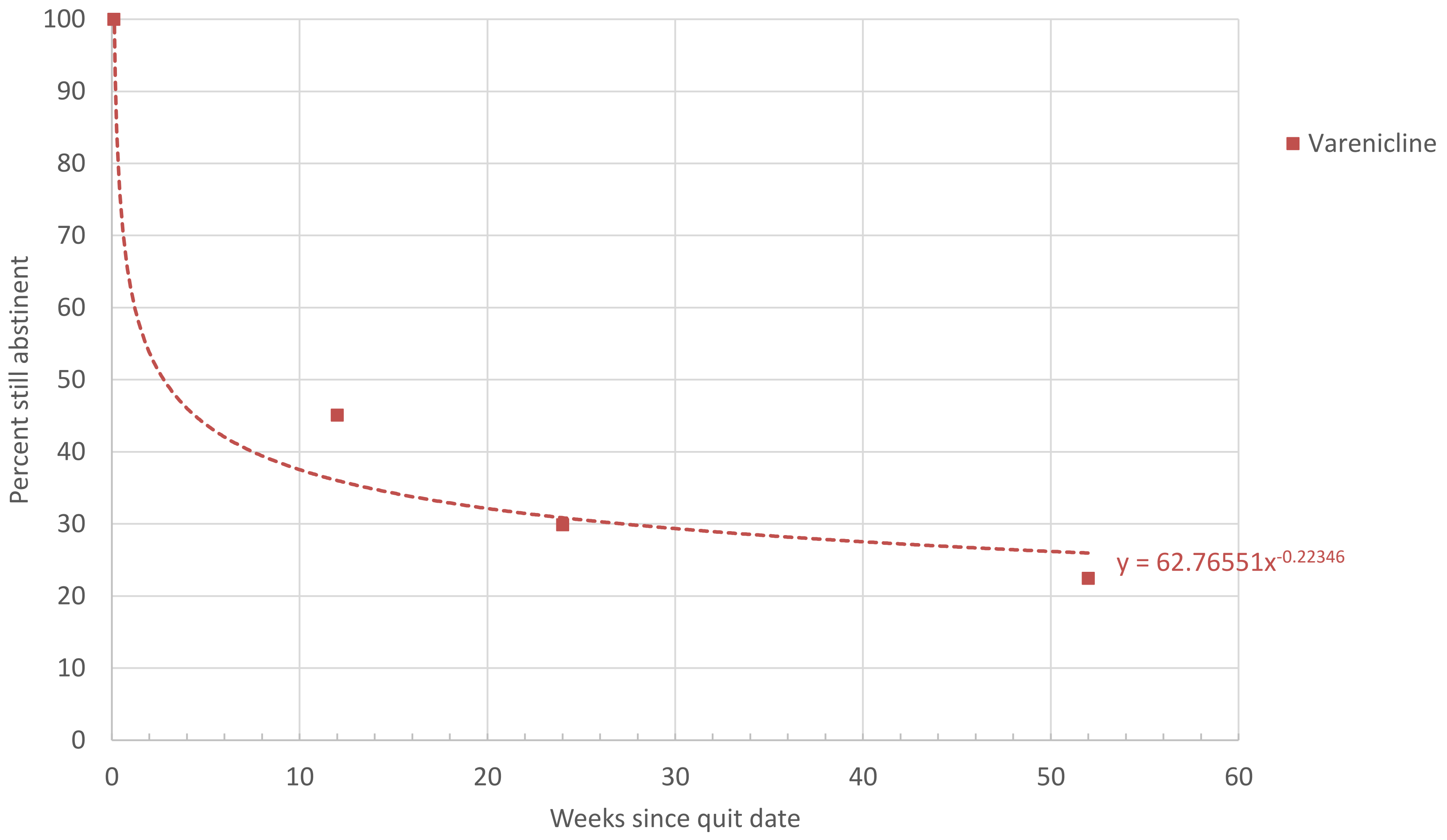

Supplementary Figure 2. Continuous abstinence rates with best-fit curves estimated from continuous abstinence rates from RCTs of 12-week treatment with (A) bupropion, (B) varenicline, (C) nicotine replacement therapy and (D) placebo, with consecutive pairs of data points from the three largest studies for each treatment

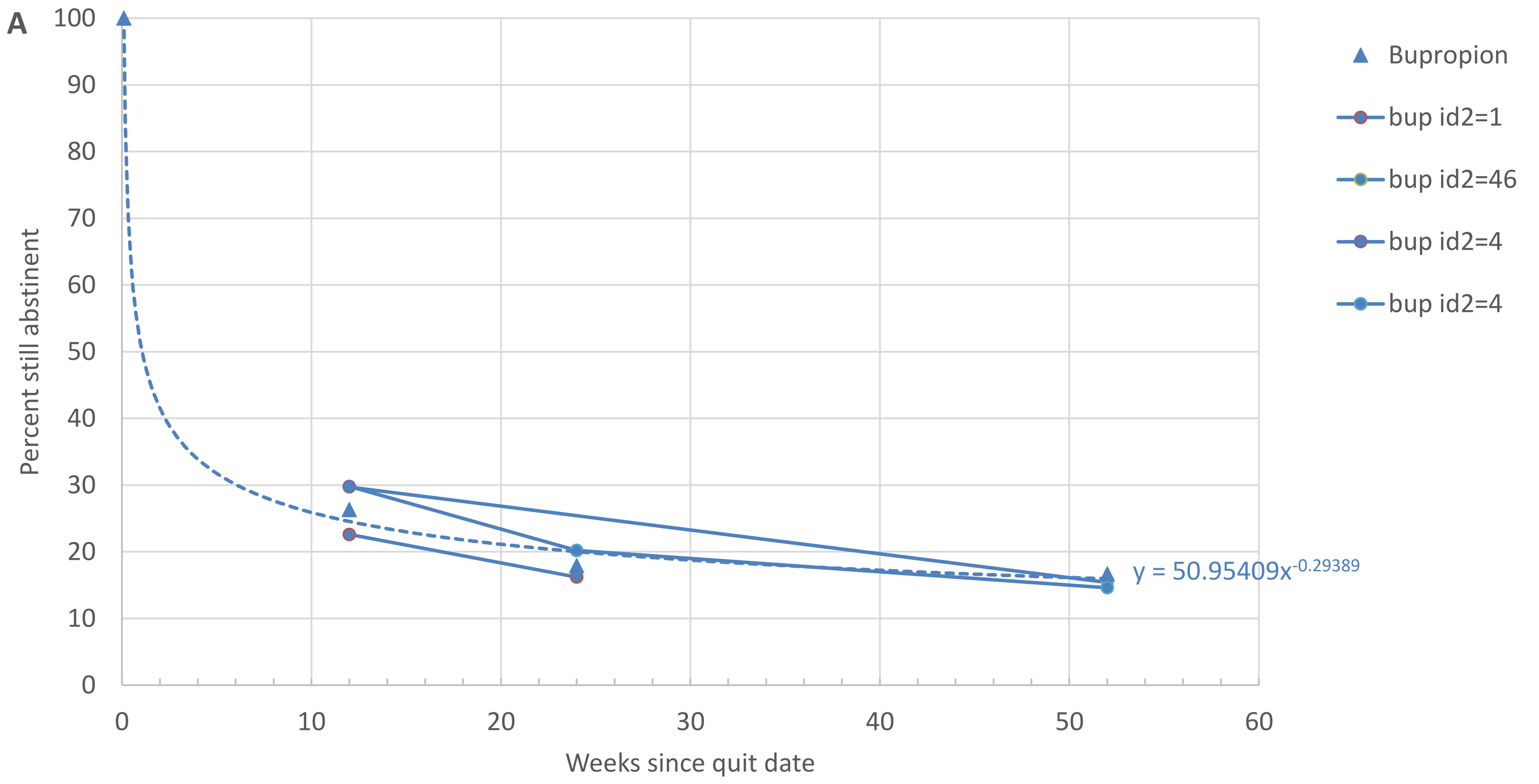

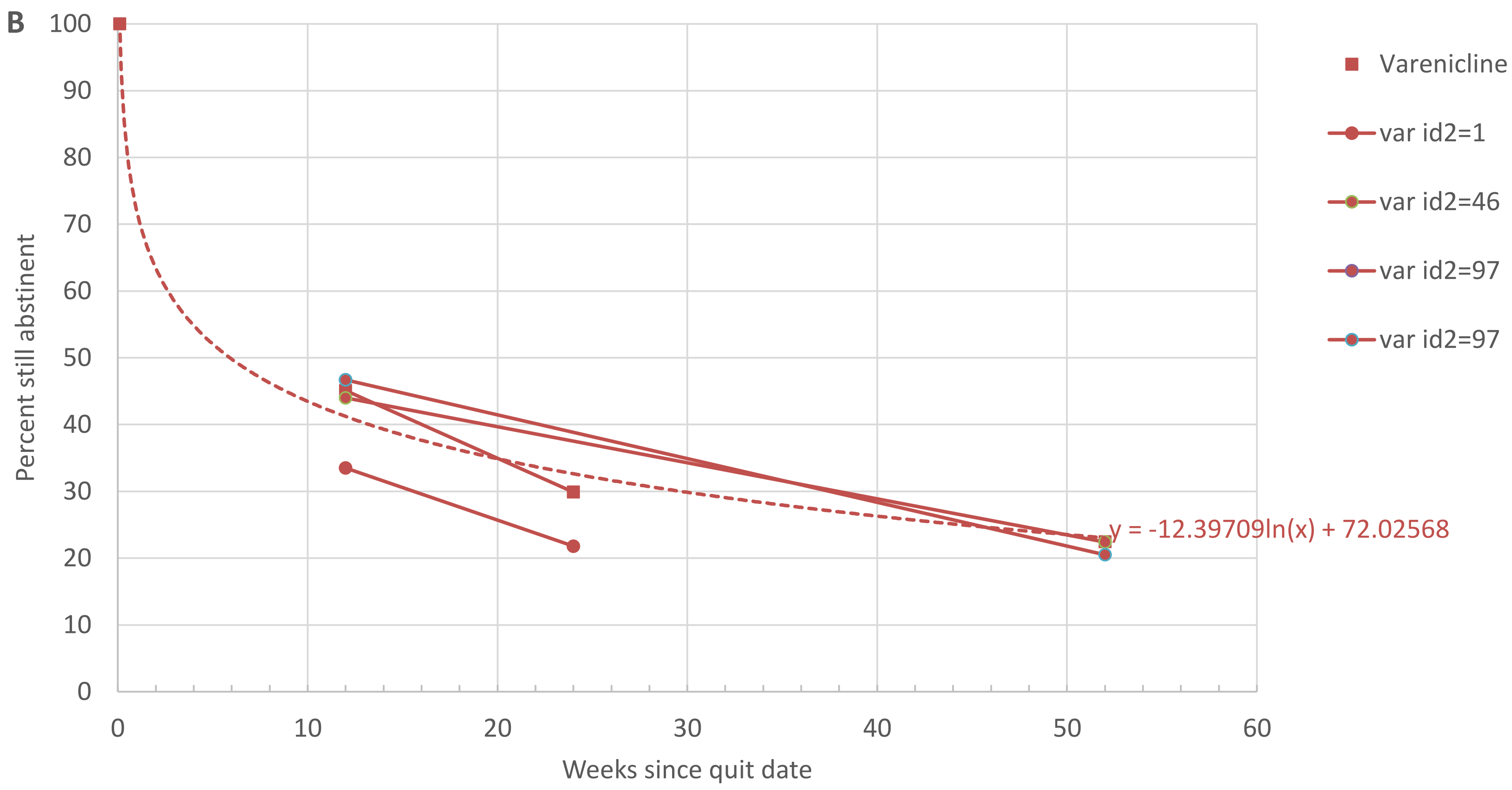

B
Percent still abstinent
Weeks since quit date
100
90
80
70
60
50
40
30
20
10
0
0
10
20
30
40
50
60
y = -12.39709ln(x) + 72.02568
Varenicline
var id2=1
var id2=46
var id2=97
var id2=97

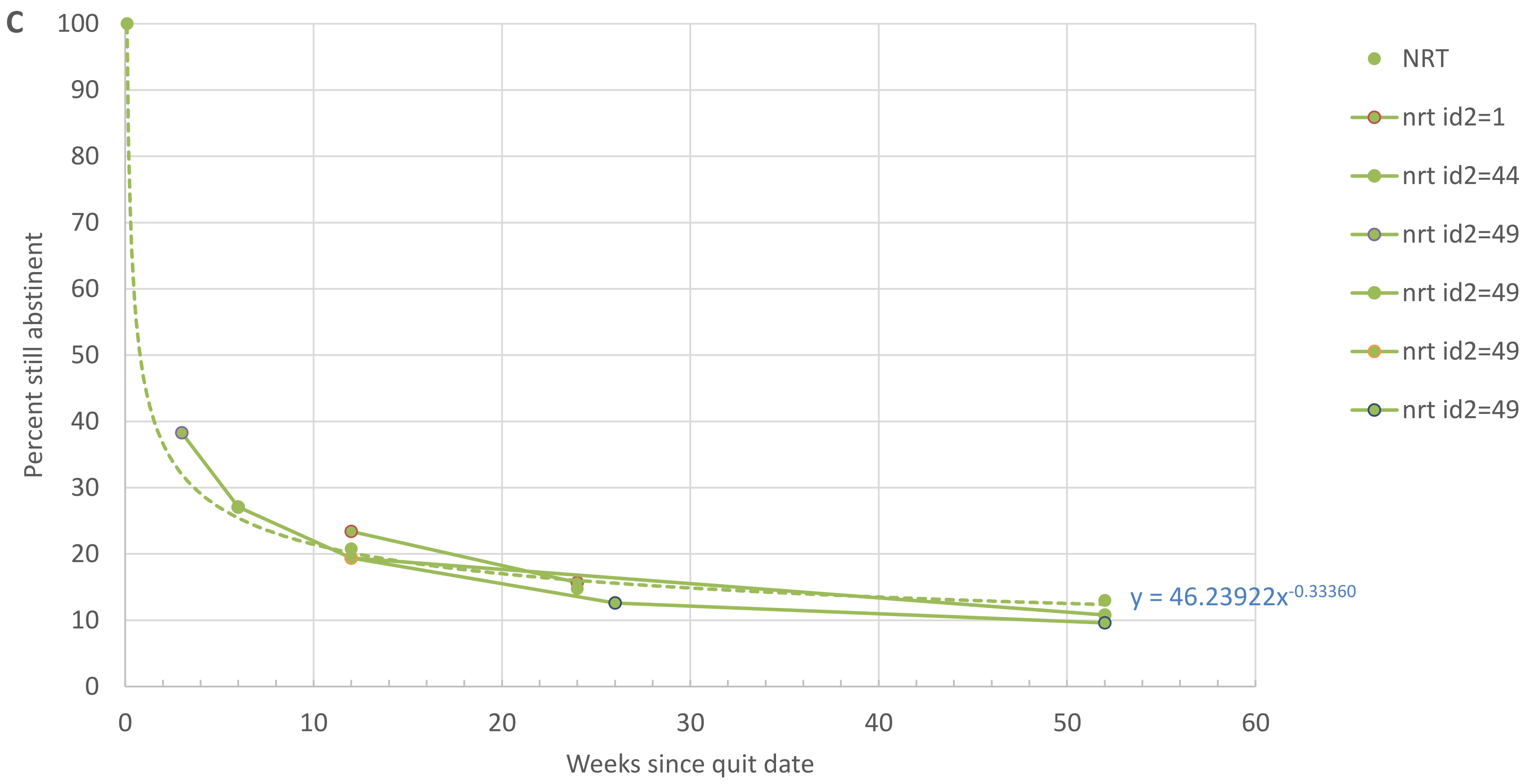
C
100
90
80
70
60
50
40
30
20
10
0
Percent still abstinent
0
10
20
30
40
50
60
Weeks since quit date
y = 46.23922x-0.33360
NRT
nrt id2=1
nrt id2=44
nrt id2=49
nrt id2=49
nrt id2=49
nrt id2=49

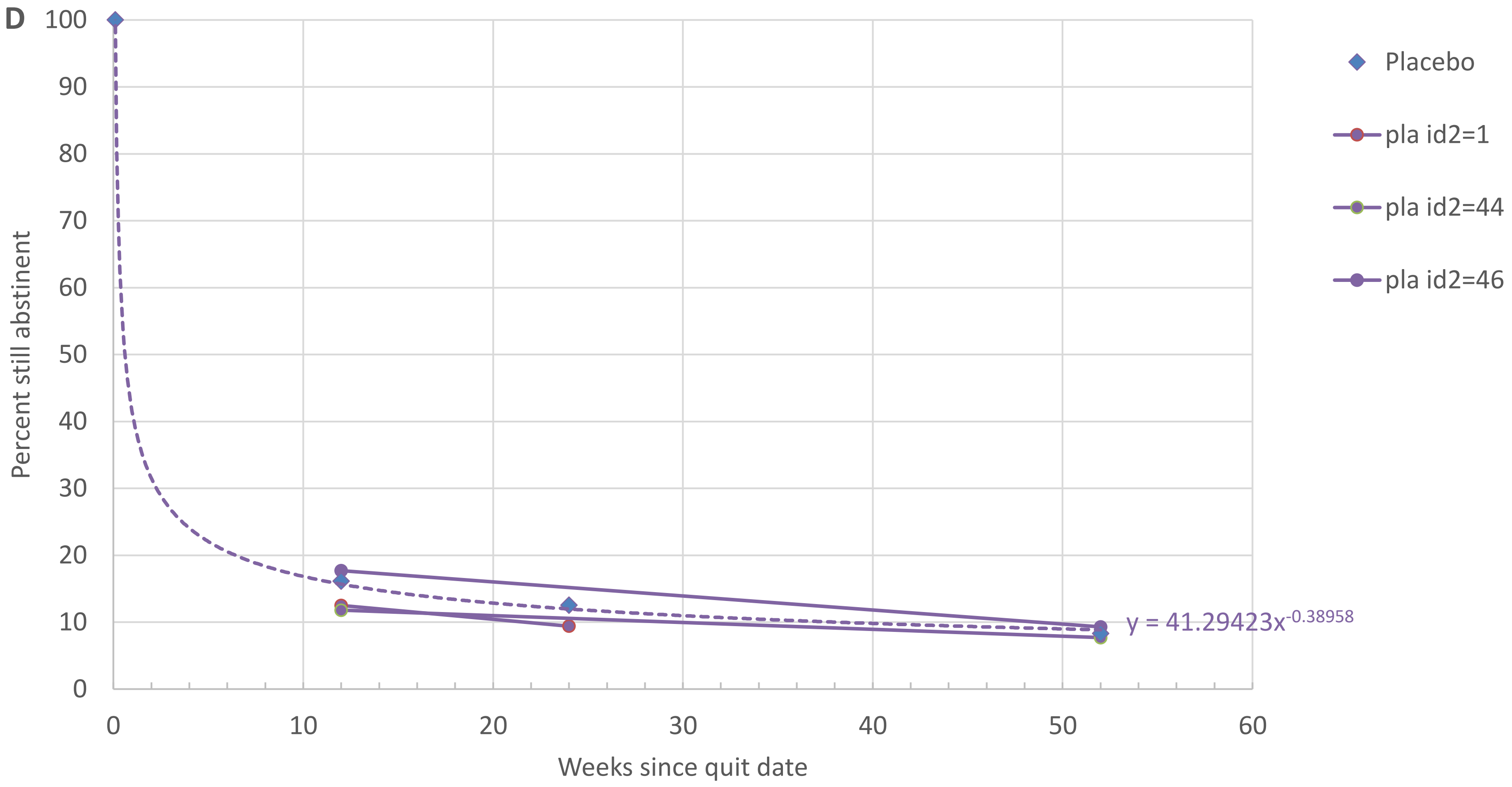

D
Percent still abstinent
100
90
80
70
60
50
40
30
20
10
0
0
10
20
30
40
50
60
Weeks since quit date
y = 41.29423x^-0.38958
Placebo
pla id2=1
pla id2=44
pla id2=46
